# T-cell activation Rho GTPase-activating protein maintains intestinal homeostasis by regulating intestinal T helper cells differentiation through the gut microbiota

**DOI:** 10.3389/fmicb.2022.1030947

**Published:** 2023-01-10

**Authors:** Ruirui He, Jianwen Chen, Ziyan Zhao, Changping Shi, Yanyun Du, Ming Yi, Lingyun Feng, Qianwen Peng, Zhihui Cui, Ru Gao, Heping Wang, Yi Huang, Zhi Liu, Chenhui Wang

**Affiliations:** ^1^The Key Laboratory for Human Disease Gene Study of Sichuan Province and the Department of Laboratory Medicine, Sichuan Provincial People's Hospital, University of Electronic Science and Technology of China, Chengdu, China; ^2^Research Unit for Blindness Prevention of the Chinese Academy of Medical Sciences, Sichuan Academy of Medical Sciences and Sichuan Provincial People's Hospital, Chengdu, Sichuan, China; ^3^Key Laboratory of Molecular Biophysics of the Ministry of Education, Hubei Key Laboratory of Bioinformatics and Molecular-imaging, Department of Biotechnology, College of Life Science and Technology, Huazhong University of Science and Technology, Wuhan, Hubei, China; ^4^Key Laboratory of Molecular Biophysics of the Ministry of Education, National Engineering Research Center for Nanomedicine, College of Life Science and Technology, Huazhong University of Science and Technology, Wuhan, China

**Keywords:** TAGAP, *Akkermansia muciniphila*, *Bacteroides acidifaciens*, Th17, colitis

## Abstract

Common variants of the T-cell activation Rho GTPase-activating protein (TAGAP) are associated with the susceptibility to human inflammatory bowel diseases (IBDs); however, the underlying mechanisms are still unknown. Here, we show that TAGAP deficiency or TAGAP expression downregulation caused by TAGAP gene polymorphism leads to decreased production of antimicrobial peptides (AMPs), such as reg3g, which subsequently causes dysregulation of the gut microbiota, which includes *Akkermansia muciniphila* and *Bacteroides acidifaciens* strains. These two strains can polarize T helper cell differentiation in the gut, and aggravate systemic disease associated with the dextran sodium sulfate-induced (DSS) disease’s phenotype in mice. More importantly, we demonstrated that recombinant reg3g protein or anti-p40 monoclonal antibody exerted therapeutic effects for the treatment of DSS-induced colitis in wild-type and TAGAP-deficient mice, suggesting that they are potential medicines for human IBD treatment, and they may also have a therapeutic effect for the patients who carry the common variant of TAGAP rs212388.

## Introduction

Inflammatory bowel diseases (IBDs), which include Crohn’s disease (CD) and ulcerative colitis (UC), are characterized by chronic recurring inflammation of the gastrointestinal tract and affect 0.5% of population worldwide (Molodecky et al., 2012; Neurath and Travis, 2012). These two diseases exhibit distinct pathophysiological features. In UC, inflammation is limited to the colon, and occurs in a continuous pattern involving the superficial mucosal and submucosal layers (de Souza and Fiocchi, 2016). In contrast, inflammation in CD appears to be discontinuous and can affect any region of the gut. The precise mechanism underlying IBD is largely elusive; however, accumulating evidence suggests that several factors contribute to IBD initiation and progression, including host genetic factors, environmental exposure, and aberrant changes in the gut microbiota composition. Patients with long-standing UC and CD have an increased risk of developing colorectal cancer and patients with small intestinal CD are at increased risk of small bowel adenocarcinoma (Eaden et al., 2001; Feagins et al., 2009).

Commensal intestinal bacteria influence their host’s metabolism and physiology in multiple ways, and they also profoundly affect the host immune system (Hooper and Gordon, 2001; Macpherson and Harris, 2004). For example, the severity of DSS-induced colitis relies heavily on intestinal microbiota, whose metabolites reportedly influence systemic immune responses (Forster et al., 2022). The microbiota composition differs in different disease stages, or in an individual or mouse of a certain genetic background, leading to a situation termed as dysbiosis (Lozupone et al., 2012; de Souza and Fiocchi, 2016). For example, the gut microbiota composition of patients with IBD is reportedly altered compared to those of healthy individuals, indicating reduced diversity and increase in mucosa-adherent bacteria (Manichanh et al., 2012; Pascal et al., 2017). The reason underlying the change in microbiota composition of patients with IBD is still unclear; however, it has been proposed that these compositional changes in the microbiota are responsible for switching the response of tolerance that normally occurs in healthy individuals, to an potentially pathogenic, immune response in IBD patients (Manichanh et al., 2012). Moreover, it was found that specific bacterial species can prime T cells toward certain directions, such as Th17 cell priming by the segmented filamentous bacterium (SFB) and Th1 cell priming by *Klebsiella pneumoniae* (Gaboriau-Routhiau et al., 2009; Ivanov et al., 2009; Atarashi et al., 2017). Once primed, T helper cells can further contribute to disease pathogenesis (Simpson et al., 1998). Recently, *Akkermansia muciniphila*, an intestinal bacterium associated with systemic effects on host metabolism and PD-1 checkpoint immunotherapy, was found to be associated with several different diseases, such as metabolic syndrome and IBD (Dao et al., 2016; Routy et al., 2018). Interestingly, *A. muciniphila* was considered to be a beneficial bacterial species, as it exerted positive treatment effects for many diseases showed the ability to prime the Th1 cell population in humans (Dao et al., 2016; Cekanaviciute et al., 2017; Plovier et al., 2017; Matson et al., 2018; Routy et al., 2018). *A. muciniphila* was reportedly absent from patients with early-onset CD (Gobert et al., 2016; Li et al., 2016), while another group found that *A. muciniphila* abundance was increased in CD patients (Danilova et al., 2019); One report found that oral gavage of *A. muciniphila* aggravates colitis for IL-10-deficient mouse, while another study did not find the same phenotype (Seregin et al., 2017; Ring et al., 2019). Therefore, it is still unclear whether it’s involved in IBD pathogenesis.

T-cell activation Rho GTPase-activating protein (TAGAP) is associated with the susceptibility to many autoimmune diseases, including IBD, multiple sclerosis, psoriasis, rheumatoid arthritis and celiac disease (Smyth et al., 2008; Raychaudhuri et al., 2009; Franke et al., 2010; Tsoi et al., 2012), based on a number of genomic studies investigating the effects of single nucleotide polymorphisms (SNPs) near or within the TAGAP gene locus. Recently, we found that TAGAP plays a critical role in anti-fungal innate immune signaling pathway, and indirectly regulates peripheral Th17 cells differentiation. Individuals who carry multiple sclerosis (MS)-associated TAGAP polymorphism show deregulated Th17 and Th1 cell abundance in the peripheral blood mononuclear cells (PBMCs), which partially explain the mechanism of TAGAP polymorphism to multiple sclerosis (MS) susceptibility (Chen et al., 2020). However, whether TAGAP is involved in intestinal mucosal immunity is still unknown. A common SNP of TAGAP is rs212388, whose “C” residue is highly associated with susceptibility to IBD (Connelly et al., 2012, 2014). Approximately 36.7% of Europeans, 57% of Asians and over 50% of people worldwide carry this polymorphism.^1^ Here, we report that TAGAP-deficient mice are susceptible to DSS-induced colitis, and this is due to significantly increased abundance of IL-17A-and IFN-γ-producing colitogenic CD4^+^ T cells in the gut. We also provide evidence showing that the gut microbiota is responsible for the dysregulation of T helper cells in TAGAP-deficient mice, and fecal transplantation of gut microbiota from wild-type mice can reverse the severity of the colitis phenotype in TAGAP-deficient mice. Moreover, by 16S rDNA sequencing, we identified several bacterial species differing in abundance between control mice and TAGAP-deficient mice. Among these species, *A. muciniphila* was much reduced in abundance in the gut of TAGAP-deficient mice compared to control mice, while the abundance of strain *Bacteroides acidifaciens* was greatly increased in TAGAP-deficient mice. Both *A. muciniphila* and *B. acidifaciens* can polarize colitogenic CD4^+^ T cell *in vivo*; and both aggravated DSS-induced systemic disease. When TAGAP was deficiency or TAGAP gene had UC-susceptible polymorphism, AMPs such as reg3g expression was greatly reduced, which is a possible reason for the dysregulation of gut microbiota in TAGAP-deficient mice. Importantly, oral feeding of reg3g recombinant protein or intraperitoneal injection of anti-p40 monoclonal antibody greatly attenuated the DSS-induced systemic disease phenotype in wild-type and TAGAP-deficient mice, which suggests that both methods may be effective for the treatment of patients with IBD, whether or not the patients who carry the IBD-susceptible TAGAP polymorphism rs212388.

## Results

### T-cell activation Rho GTPase-activating protein deficiency exacerbates colitis severity

**Figure 1:**
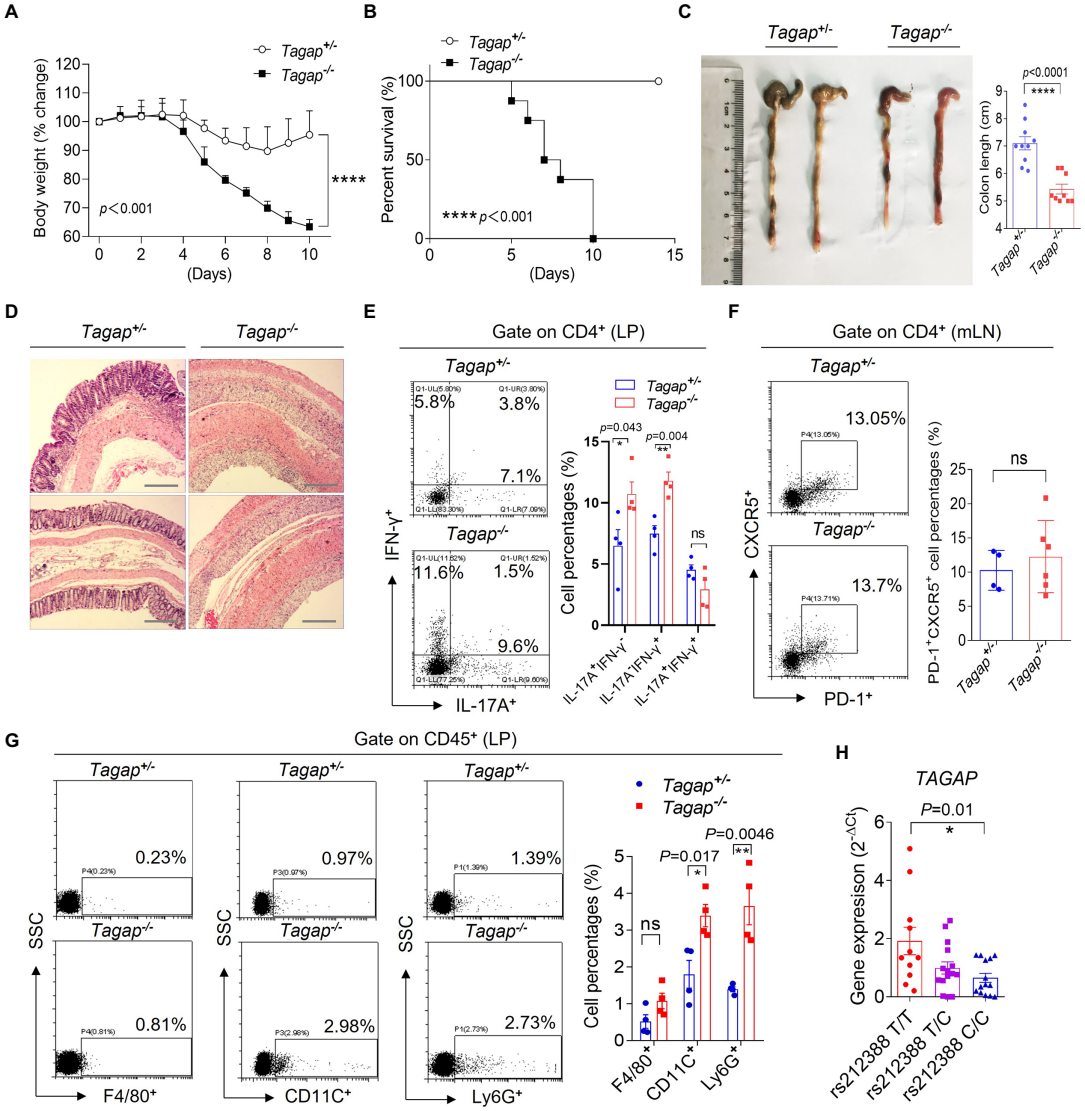
TAGAP-deficient mice were susceptible to DSS-induced colitis. **(A)** Littermate control mice or TAGAP-deficient mice were treated with 3% of DSS in the drinking water for 5 days, and then changed to normal water. Mice weight curve was shown, *n* = 7. **(B)** Littermate control mice or TAGAP-deficient mice were treated with 3% of DSS in the drinking water for 5 days, and then changed to normal water. Mice survival curve was shown, *n* = 9. **(C)** Representative colon picture of DSS-treated control mice and TAGAP-deficient mice was shown (left). Colon length of control mice and TAGAP-deficient mice after DSS treatment was shown (right), *n* = 9. **(D)** Hematoxylin staining of transversal sections of colon sample from DSS-treated control mice and TAGAP-deficient mice at day 7 was shown. Scale bar: 150 μm. **(E–G)** Cells were isolated from lamina propria **(E,G)** or mesenteric lymph nodes **(F)** from littermate control mice or TAGAP-deficient mice after DSS treatment for 5 days, followed by flow cytometry analysis of indicated cells, *n* = 4. **(H)** *TAGAP* mRNA level was examined in the PBMCs of different individuals who carried the indicated *TAGAP* genotypes, *n* = 11, 15, and 14. The weight loss **(A)** and survival data **(B)** were from separate experiments. *p < 0.05, **p < 0.01, ***p < 0.001, ****p < 0.0001 based on two-way ANOVA **(A)**, Two-sided unpaired T test (**C,E–H**) and Log-rank (Mantel-Cox) Test for panel (**B**). All error bars represent SEM of technical replicates. Data are representative of three independent experiments.

The TAGAP gene locus has been associated with several inflammatory diseases, including Crohn’s disease (Connelly et al., 2014). To investigate the role of TAGAP in the pathogenesis of IBD, we tested how genetic deletion of TAGAP impacts colitis severity in the DSS colitis model. Mice with homozygous deletion of TAGAP (*Tagap^−/−^*) and their heterozygous littermate controls (*Tagap^+/−^*) were housed independently following weaning, and at 12 weeks of age received DSS in the drinking water for a total of 5 days. Mice were then monitored daily for weight loss and survival. We found that *Tagap^−/−^* mice had significantly greater weight loss within 10 days of DSS administration, while *Tagap^+/−^* mice had more modest weight loss that abated by 3 days following DSS withdrawal (Figures 1A,B). Gross examination revealed that colon length was significantly decreased in *Tagap^−/−^* mice, and histopathologic examination demonstrated pathologic hallmarks of severe colitis, including epithelial disruption, crypt dropout, and transmural mononuclear cell infiltration, which were not seen in *Tagap^+/−^* controls (Figures 1C,D).

CD4^+^ T cells have been shown to play an important immunoregulatory role in the gut, and the homeostatic balance between inflammation-constraining and colitogenic T cell populations is thought to be a key determinant of colitis disease activity. In particular, studies examining mouse and human colonic tissue have reported on IL-17A^+^ CD4^+^ T cell or IFNγ^+^IL-17A^+^ CD4^+^ T cell populations that has been shown to exacerbate colitis severity (Cosmi et al., 2008; Ahern et al., 2010; Chudnovskiy et al., 2016; Britton et al., 2019; Schirmer et al., 2019). Successful clinical trials employing IL-12/IL-23 inhibition have further underlined the importance of T cells in the pathogenesis of at least some patients with IBD. We explored whether CD4^+^ T cells were important mediators of the severe colitis phenotype observed in TAGAP deficiency. Strikingly, colonic lamina propria (LPs) of *Tagap^−/−^* mice had significantly increased IL-17A-producing and IFN-γ-producing CD4^+^ T cells compared to that in control mice, while the Tfh cells in the mesenteric lymph nodes (mLN) did not have difference (Figures 1E,F). IL-17A and IFN-γ-producing CD4^+^ T cells were known to induce local inflammation by recruiting inflammatory cells in the IBD (Viladomiu et al., 2017; Omenetti et al., 2019). Strikingly, we found that the infiltrated CD11C^+^ cells and Ly6G^+^ cells were significantly increased in the colonic lamina propria of *Tagap^−/−^* mice compared to control mice (Figure 1G). Notably, although *Tagap^−/−^* mice were smaller and less weight compared to control mice, *Tagap^−/−^* mice and *Tagap^+/−^*mice that did not receive DSS had no significant difference in inflammatory gene expression evidence of colitis (Supplementary Figures S1A,B). There wasn’t significant difference in terms of IL-17A or IFNγ-producing CD4^+^ T cells in the colon of *Tagap^−/−^* mice and control mice without treatment of DSS (Supplementary Figure S1C). We also detected Treg cells in the colonic lamina propria from colitis mice, and found that there was no significant difference between *Tagap^+/−^* control mice and *Tagap^−/−^* mice (Supplementary Figure S1D). Together, these data indicate that following mucosal injury, TAGAP deficiency was associated with increased IL-17A-producing and IFN-γ-producing CD4^+^ T cells infiltration and proinflammatory gene expression, which greatly exacerbated colitis severity.

The intronic polymorphism rs212388 (T- > C), which is located in the predicted promoter region of TAGAP, was found in genome wide association studies to be associated with increased IBD susceptibility (Connelly et al., 2012, 2014). Given that genetic deletion of TAGAP markedly exacerbated colitis severity in the DSS mouse model, we hypothesized that rs212388 might confer increased IBD risk by modulating TAGAP gene expression. To test this hypothesis, we measured TAGAP mRNA levels in peripheral blood mononuclear cells (PBMCs) from human volunteers with wild-type (T/T), heterozygous (T/C) and homozygous (C/C) genotype at the rs212388 polymorphism. Interestingly, rs212388 (C/C) PBMCs had significantly lower levels of TAGAP mRNA when compared to either rs212388 (T/T) or rs212388 (T/C) PBMCs (Figure 1H). The human PBMC data is consistent with mice data that decreased TAGAP expression exacerbated colitis severity (Figures 1A–D).

### Colitis severity in *Tagap^−/−^* mice is dependent on the microbiota

**Figure 2:**
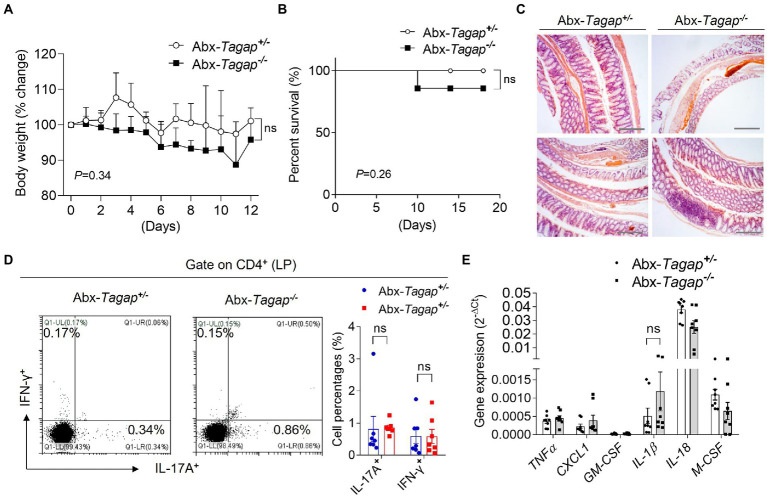
Clearance of gut microbiota attenuated DSS-induced colitis severity for TAGAP-deficient mice. **(A,B)** Littermate control mice or TAGAP-deficient mice were pretreated antibiotics for 2 weeks as described in the Method, followed by 3% of DSS treatment for 7 days, and then changed to normal water. Mice weight curve **(A)** and survival curve **(B)** were done in separate experiments, *n* = 9. **(C)** Mice were treated as in A, and hematoxylin staining of transversal sections of colon sample at day 7 was shown. Scale bar: 150 μm. **(D)** Mice were treated as in A, and cells were isolated from lamina propria of control mice or TAGAP-deficient mice after DSS treatment for 5 days, followed by flow cytometry analysis of CD4^+^IL-17A^+^ and CD4^+^IFN-γ^+^ cells, *n* = 7. **(E)** Mice were treated as in A, and colonic samples were isolated from control mice or TAGAP-deficient mice, followed by real-time PCR analysis for the indicated genes, *n* = 8. *p < 0.05, **p < 0.01, ***p < 0.001, ****p < 0.0001 based on two-way ANOVA **(A)** and Log-rank (Mantel-Cox) Test for panel **(B)**. All error bars represent SEM of technical replicates. Data are representative of three independent experiments.

The gut microbiome has been shown to play an important role in the pathogenesis of IBD. Accumulating evidence suggests that this role is mediated, at least in part, by the capacity of microbiota to shape the gut immune environment (Gaboriau-Routhiau et al., 2009; Ivanov et al., 2009; Atarashi et al., 2017). Furthermore, several bacterial strains have been shown to skew colonic CD4^+^ T cell populations toward proinflammatory effector phenotypes. To test whether gut microbiota play any role in the severe colitis phenotype seen in *Tagap^−/−^* mice, we depleted the bacterial communities of *Tagap^−/−^* mice and controls using broad-spectrum antibiotics. After confirming microbiota clearance, we treated mice with DSS. In contrast to our prior experiments, there was no significant difference in weight loss or survival between *Tagap^−/−^* and *Tagap^+/−^* mice following microbiota depletion (Figures 2A,B). Histopathologic analysis of colon sections also showed comparable colitis severity between groups (Figure 2C). Furthermore, colitogenic T cell populations from the colonic lamina propria were not significantly different between *Tagap^−/−^* mice and controls (Figure 2D). Proinflammatory gene expression in colonic tissue from *Tagap^−/−^* and control mice was also similar (Figure 2E). Together, these results indicated that the increased expression of proinflammatory genes, the accumulation of colitogenic CD4^+^ T cells, and the development of severe colitis in TAGAP-deficient mice is dependent on the microbiota.

### Fecal microbiota transplantation attenuates colitis severity in *Tagap^−/−^* mice

**Figure 3:**
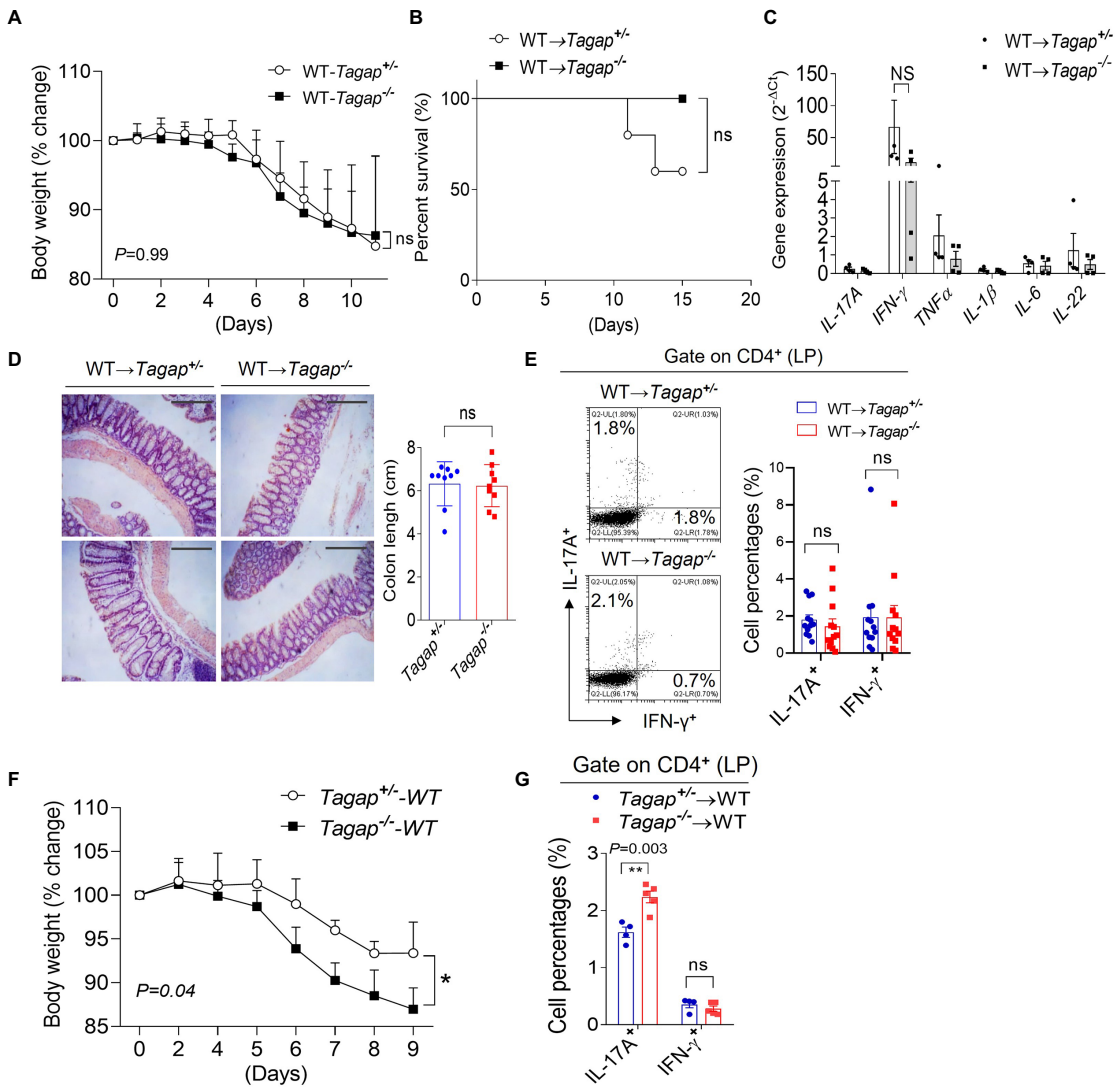
FMT rescue the severe phenotype of TAGAP-deficient mice in colitis. **(A,B)** Littermate control mice or TAGAP-deficient mice were transplanted feces from wild-type mice as described in the Method, followed by 3% of DSS treatment for 5 days, and then changed to normal water. Mice weight curve was shown in panel **(A)**, *n* = 9; mice survival curve was shown in panel **(B)**, *n* = 5. Mice weight curve **(A)** and survival curve **(B)** were done in separate experiments. **(C)** Mice were treated as in A, and colonic samples were isolated from littermate control mice or TAGAP-deficient mice after DSS treatment for 7 days, followed by real-time PCR analysis for the indicated genes. **(D)** Mice were treated as in A, and hematoxylin staining of transversal sections of colon sample after DSS treatment for 10 days was shown (left panel), Scale bar: 150 μm. Colon length was shown (right panel), *n* = 9. **(E)** Mice were treated as in A, and cells were isolated from lamina propria of control mice or TAGAP-deficient mice after DSS treatment for 5 days, followed by flow cytometry analysis of CD4^+^IL-17A^+^ and CD4^+^IFN-γ^+^ cells, *n* = 12. **(F)** Wild-type mice were transplanted feces from littermate control mice or TAGAP-deficient mice as described in the Method, followed by 3% of DSS treatment for 5 days, and mice weight change was shown, *n* = 4. **(G)** Mice were treated as in F, and cells were isolated from lamina propria of wild-type recipient mice after DSS treatment for 5 days, followed by flow cytometry analysis of CD4^+^IL-17A^+^ and CD4^+^IFN-γ^+^ cells, *n* = 5. *p < 0.05, **p < 0.01, ***p < 0.001, ****p < 0.0001 based on 2-way ANOVA **(A,F)**, Log-rank (Mantel-Cox) Test for **(B)** and unpaired T test **(C–E,G)**. All error bars represent SEM of technical replicates. Data are representative of three independent experiments.

A number of preclinical studies have suggested that fecal microbiota transplantation (FMT) holds promise as a potential therapeutic modality in a number of diseases. The effectiveness of FMT has already been established in clinical trials for the treatment of recalcitrant *Clostridioides difficile* infections (Bakken et al., 2011; Borody and Khoruts, 2011). Because we had found that the severe colitis phenotype in TAGAP deficiency is abolished following depletion of the microbiota, we wondered whether FMT might be sufficient to rescue the severe colitis phenotype. Indeed, we found that feces from wild-type donor mice rescued the severe phenotype of *Tagap^−/−^* mice during DSS-induced colitis, as measured by both weight loss and survival (Figures 3A,B). Moreover, colons from *Tagap^−/−^* and *Tagap^+/−^* control mice expressed comparable levels of proinflammatory cytokine genes (Figure 3C). Analysis of whole colons and colon sections showed similar colon length and histopathologic severity between *Tagap^−/−^* mice and controls. Interestingly, following FMT *Tagap^−/−^* mice actually had fewer colitogenic T cells in the colonic lamina propria than *Tagap^+/−^* controls (Figures 3D,E). These findings suggest that the protective effect of TAGAP signaling in colitis is transferrable *via* the microbiota. We therefore wondered whether, conversely, the exacerbation of colitis severity seen in TAGAP deficiency might also be transferrable. To our surprise, we found that the transfer of *Tagap^−/−^* feces to wild-type mice indeed recapitulated the severe colitis phenotype seen in *Tagap^−/−^* mice (Figure 3F). Consistent with this finding, the colonic lamina propria of mice receiving *Tagap^−/−^* feces contained significantly increased numbers of colitogenic CD4^+^ T cells (Figure 3G). Cumulatively, these data suggested that the severe colitis phenotype seen in TAGAP deficiency is critically mediated by the gut microbiota, is transferable *via* FMT, and is associated with increased numbers of colitogenic CD4^+^ T cells.

### T-cell activation Rho GTPase-activating protein deficiency leads to gut dysbiosis and immune dysregulation in the colitis model

**Figure 4:**
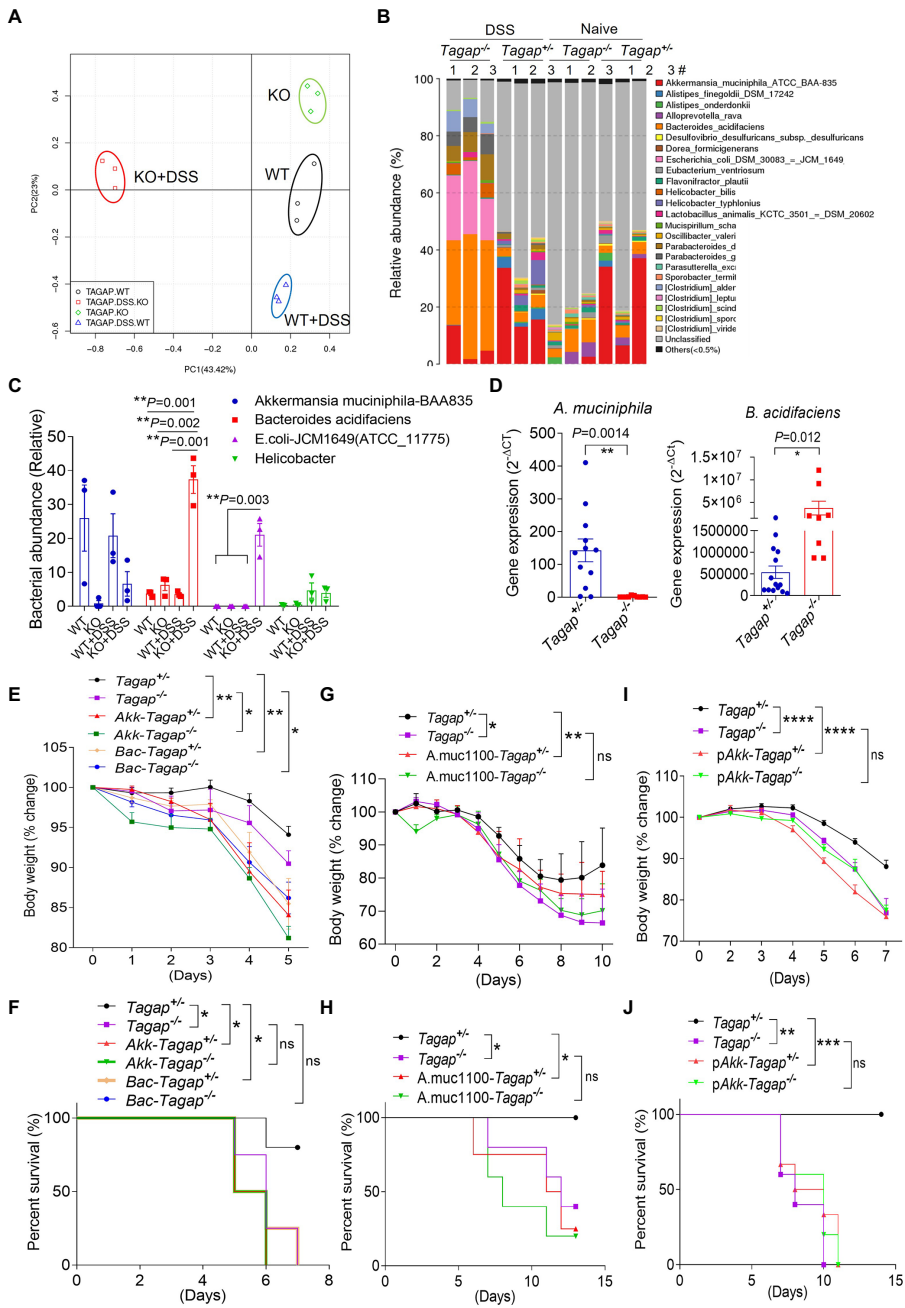
TAGAP-deficient mice had altered microbiota in the gut. **(A,B)** Cluster analysis or diversity analysis of overall gut microbiota in the littermate control mice or TAGAP-deficient mice, *n* = 3. **(C)** Relative abundance of different bacterial strain in the feces of littermate control mice or TAGAP-deficient mice identified by 16S sequencing was shown, *n* = 3. **(D)** Feces from littermate control mice or TAGAP-deficient mice were collected and analyzed by real time PCR for the indicated bacterial strains, *n* = 12. **(E,F)** Littermate control mice or TAGAP-deficient mice were oral gavaged *Akkermansia muciniphila* or *Bacteroides acidifaciens* daily for 4 weeks (1 × 10^8^/mouse/day), followed by 2.5% of DSS treatment for 5 days, and then changed to normal water. The weight loss **(E)** and survival data **(F)** were from separate experiments. **(G,H)** Littermate control mice or TAGAP-deficient mice were oral gavaged recombinant Amuc_1100 protein daily for 4 weeks (10 μg/mouse/day), followed by 2.5% of DSS treatment for 5 days. The weight loss **(G)** and survival data **(H)** were from separate experiments. **(I,J)** Littermate control mice or TAGAP-deficient mice were oral gavaged pasteurized *A. muciniphila* daily for 4 weeks (1 × 10^8^/mouse/day), followed by 2.5% of DSS treatment for 5 days. The weight loss **(I)** and survival data **(J)** were from separate experiments, *n* = 5 for panels **(E–J)**. *p < 0.05, **p < 0.01, ***p < 0.001, ****p < 0.0001 based on 2-way ANOVA **(E,G,I)**, Log-rank (Mantel-Cox) Test for panels **(F,H,J)** and unpaired T test **(D)**. Data are representative of two independent experiments.

We therefore wondered what specific changes in the microbiota of *Tagap^−/−^* mice actually underlie the induction of colitogenic T cell populations and exacerbation of colitis severity. To understand the mechanism of the severe phenotype in DSS-induced colitis model and its relationship to the increased colitogenic T cell population in TAGAP-deficient mice, we performed 16S rDNA gene sequencing of the feces from *Tagap^−/−^* mice and *Tagap^+/−^* controls. The microbiota composition of *Tagap^−/−^* mice was indeed markedly different than that of control *Tagap^+/−^* mice, and this genotype-dependent impact on microbiota composition was even more pronounced following DSS treatment (Figure 4A). Specifically, we noted that DSS treatment resulted in a dramatic reduction in microbial diversity in *Tagap^−/−^* mice, while there was minimal such impact on controls (Supplementary Figures S2B–D). The reduction in microbial diversity observed in *Tagap^−/−^* mice was associated with new prominence of several species in particular, which had been relatively minor constituents prior to DSS treatment (Figure 4B; Supplementary Figure S2A). Several of these have previously been reported to be associated with IBD in humans, including *E. coli* and *Helicobacter* species (Figures 4B,C). As the pathogenic role of *E. coli* on colitis is well-known, we focus on other bacterial strains which may have a significant influence on colitis in this study. Examination of genotype-dependent effects on microbiota composition revealed that two species in particular, *A. muciniphila* and *B. acidifaciens*, accounted for the most dramatic contrast between *Tagap^−/−^* mice and *Tagap^+/−^*controls. Whereas the abundance of *A. muciniphila* was much lower in *Tagap^−/−^* mice compared to controls, the abundance of *B. acidifaciens* was greatly increased relative to controls (Figures 4C,D).

Accumulating evidence suggests that individual bacterial species can have a significant impact on gut immune homeostasis, and in certain cases may promote a pro-inflammatory environment that exacerbates colitis severity. *A. muciniphila* has recently become the source of significant research and clinical interest, and is being investigated in clinical trials as a potential weight loss-promoting therapy (Everard et al., 2013; Matson et al., 2018; Ansaldo et al., 2019; Depommier et al., 2019). Much less is known about the functional impact of *B. acidifaciens* colonization, although another species within the genus *B. fragilis*, was previously reported to polarize colonic CD4^+^ T cells toward a pro-inflammatory IL-17A-secreting phenotype and thereby promote tumorigenesis (Wu et al., 2009). We therefore hypothesized that these species might play a role in the microbiota-dependent immune dysregulation and resultant severe colitis seen in *Tagap^−/−^* mice. To test this, we cultured *A. muciniphila* and *B. acidifaciens,* then administered these isolates *via* oral gavage to *Tagap^−/−^* and *Tagap^+/−^* control mice daily for 4 weeks. Following mucosal injury with DSS, we found that both *A. muciniphila* and *B. acidifaciens* exacerbated systemic disease’s symptom, as assessed by weight loss (Figure 4E). From the survival rate, we found that *A. muciniphila* accelerated the death rate of control mice, while did not promote the death for TAGAP-deficient mice, and this may be because of severe phenotype of TAGAP-deficient mice in the colitis model (Figure 4F). To test whether the colitogenic impact of these *A. muciniphila* and *B. acidifaciens* strains was dependent on other microbiota constituents, we depleted the gut microbiota in *Tagap^−/−^* and *Tagap^+/−^* controls using broad-spectrum antibiotics. After microbiota depletion was confirmed, we selectively colonized mice with either *A. muciniphila* or *B. acidifaciens,* then induced colitis with DSS. Consistent with our previous results, selective colonization of mice with either *A. muciniphila* or *B. acidifaciens* strains exacerbated systemic disease’s severity independent of *Tagap* genotype (Supplementary Figures S3A,B). Recent work has suggested that in the absence of live *A. muciniphila,* the outer membrane protein Amuc_1100 can induce TLR2-dependent NFκB signaling *in vitro*, and may partially mediate some reported host effects *in vivo* (Plovier et al., 2017). Therefore, we explored whether the DSS-induced systemic disease’s symptom of *A. muciniphila* in the DSS model is also recapitulated by recombinant Amuc_1100. Oral administration of recombinant Amuc_1100 did indeed aggravate DSS-induced systemic disease’s symptom in *Tagap^+/−^* mice but had no effect in *Tagap^−/−^* mice (Figures 4G,H). While this suggested that recombinant Amuc_1100 can potentiate systemic disease’s severity in a TAGAP-dependent manner, given our earlier finding that live *A. muciniphila* increases systemic disease’s severity even in TAGAP-deficient mice, these data also imply that *A. muciniphila* components other than Amuc_1100 are also capable of potentiating DSS-induced systemic disease’s severity. Recent work has demonstrated that pasteurized *A. muciniphila* is more effective at mediating host effects than recombinant Amuc_1100 alone (Depommier et al., 2019). Interestingly, we found that oral administration of pasteurized *A. muciniphila* also aggravated systemic disease’s symptom in a manner that was dependent on host TAGAP, which was consistent with what we found using Amuc_1100 supplementation (Figures 4I,J). Together, these results indicate that both *A. muciniphila* and *B. acidifaciens* are associated with gut immune dysregulation following mucosal injury and potentiate systemic disease’s severity. These data further demonstrate that while Amuc_1100 can potentiate systemic disease’s severity, *A. muciniphila* also has colitogenic potential independent of Amuc_1100.

### *Akkermansia Muciniphila* and *Bacteroides acidifaciens* promote the accumulation of Th1 and Th17 cells in the gut

**Figure 5:**
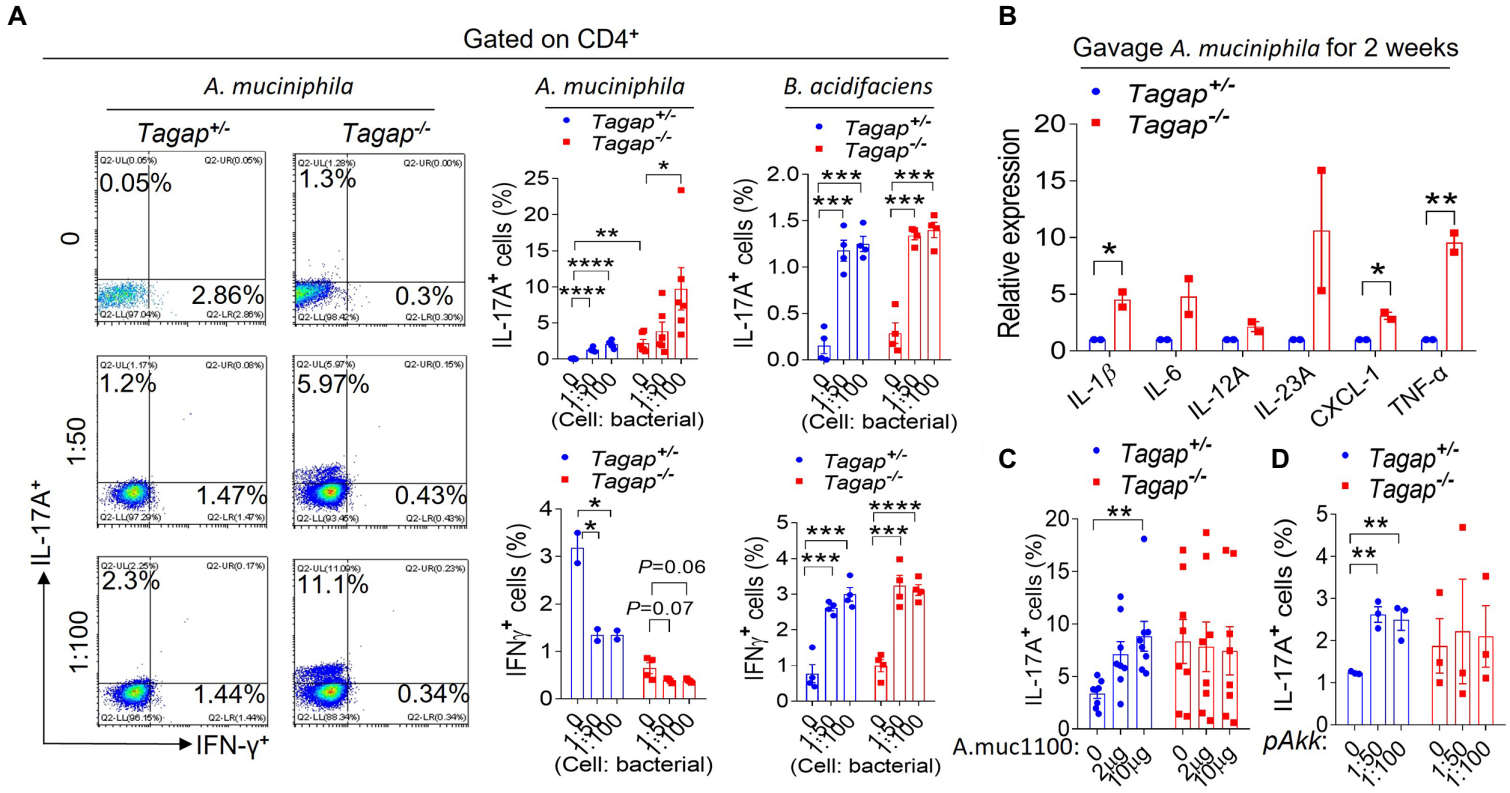
*Akkermansia muciniphila* or *Bacteroides acidifaciens* promote T helper cells polarization. **(A)** Littermate control mice or TAGAP-deficient mice were oral gavaged *A. muciniphila* twice a week for 2 weeks (1 × 10^9^), and cells were isolated from lamina propria, and incubated with *A. muciniphila* or *B. acidifaciens* for another 3 days (MOI = 50 or 100). Cells were analyzed by flow cytometry as indicated. **(B)** Littermate control mice or TAGAP-deficient mice were oral gavaged *A. muciniphila* twice a week for 2 weeks (1 × 10^9^). Colonic tissues were isolated, followed by real-time PCR analysis of indicated gene expression. **(C)** Littermate control mice or TAGAP-deficient mice were oral gavaged Amuc_1100 twice a week for 2 weeks (10 μg/mouse), and cells were isolated from lamina propria, and incubated with Amuc_1100 for another 3 days (2 μg/ml or 10 μg/ml). Cells were analyzed by flow cytometry as indicated, *n* = 8. **(D)** Littermate control mice or TAGAP-deficient mice were oral gavaged pasteurized *A. muciniphila* twice a week for 2 weeks (1 × 10^9^), and cells were isolated from lamina propria, and incubated with pasteurized *A. muciniphila* for another 3 days (MOI = 50 or 100). Cells were analyzed by flow cytometry as indicated. *p < 0.05, **p < 0.01, ***p < 0.001, ****p < 0.0001 based on unpaired T test **(C,D)**. Data are representative of three independent experiments.

Previous work by others has shown that *A. muciniphila* can promote the differentiation of CD4^+^ T cells toward an IFN-γ-producing Th1 phenotype by using human Peripheral blood mononuclear cell (PBMCs) (Cekanaviciute et al., 2017). However, to our knowledge, the impact of *B. acidifaciens* on host CD4^+^ T cell populations has not been previously examined. We administered *via* oral gavage either *A. muciniphila* or *B. acidifaciens* to *Tagap^−/−^* mice and *Tagap^+/−^* controls, after which we examined the colonic leukocyte population. Both *A. muciniphila* and *B. acidifaciens* significantly increased the accumulation of IL-17A-secreting CD4^+^ T cells. Interestingly, however, the accumulation of Th17 cells in response to *A. muciniphila* was much greater in *Tagap^−/−^* mice as compared to *Tagap^+/−^* controls, and *A. muciniphila* significantly decreased the numbers of IFNγ-secreting cells in either host genotype (Figure 5A). *B. acidifaciens* promoted a significant increase in the number of IL-17A-and IFNγ-secreting CD4^+^ T cells, and this effect was comparable between *Tagap^−/−^* mice and controls mice (Figure 5A). At the molecular level, oral administration of *A. muciniphila* induced colonic gene expression of key pro-inflammatory cytokines including (including *IL-1β, IL-6, IL-23*, and *TNFa*), to a significantly greater extent in *Tagap^−/−^* mice than in controls (Figure 5B). This result may explain the higher Th17 polarization in the gut of *Tagap^−/−^* mice as compared to *Tagap^+/−^* controls after *A. muciniphila* treatment (Figure 5A). Interestingly, oral administration of Amuc_1100 or pasteurized *A. muciniphila* promoted the accumulation of IL-17A-secreting CD4^+^ T cells in *Tagap^+/−^* mice, but not in *Tagap^−/−^* mice (Figures 5C,D). This finding mirrors our earlier results showing that Amuc_1100 and pasteurized *A. muciniphila* potentiates DSS-induced systemic disease’s severity, but in a host TAGAP-dependent manner (Figures 4G–J).

### Reg3g recombinant proteins reduces colitis severity in *Tagap^−/−^* mice

**Figure 6:**
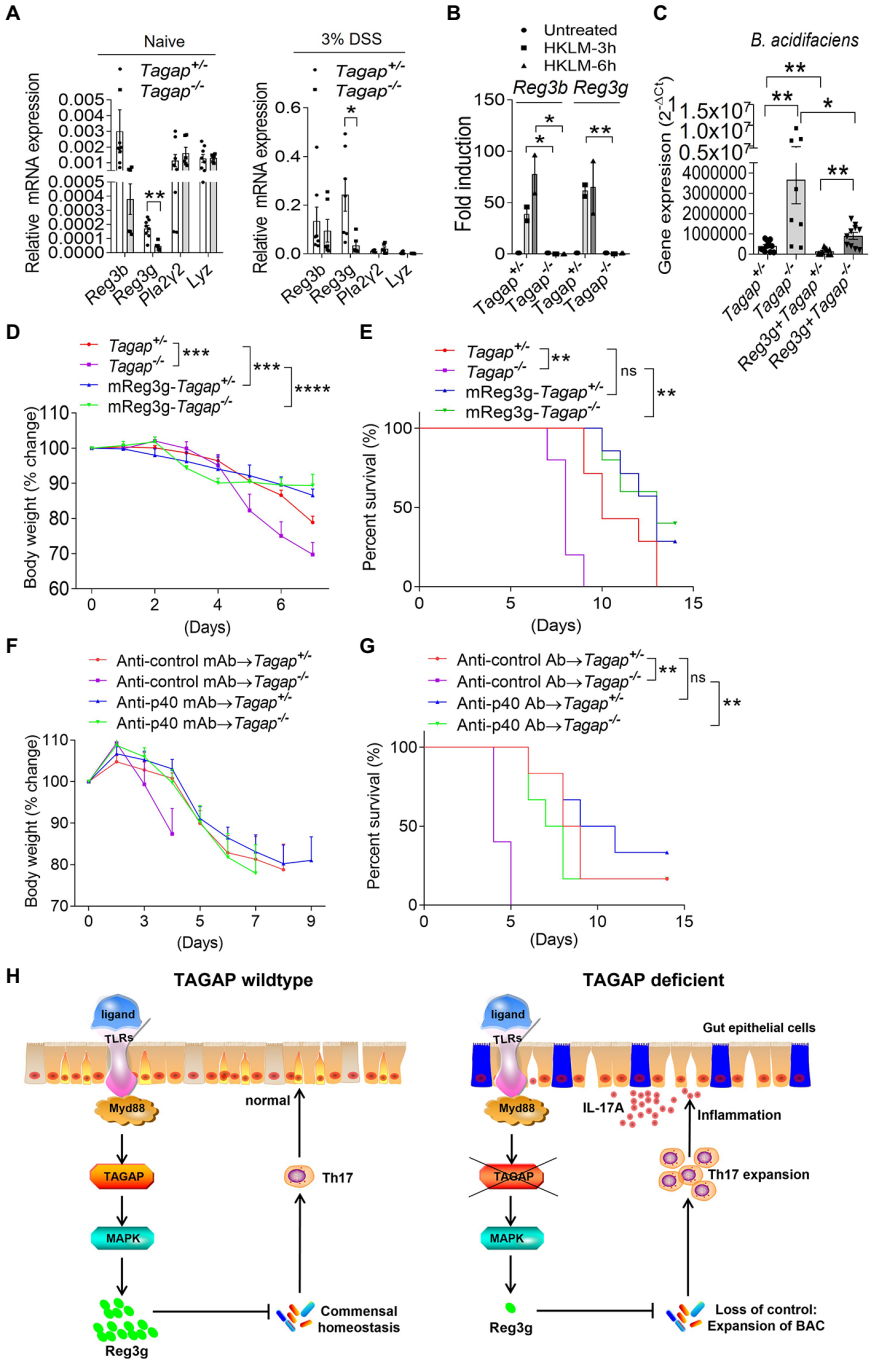
Reg3g recombinant protein or anti-p40 MAb rescue the severe phenotype of TAGAP-deficient mice in colitis. **(A)** Colonic tissues were isolated from littermate control mice or TAGAP-deficient mice with or without DSS treatment (day 5 of DSS treatment), followed by real-time PCR analysis of indicated gene expression, *n* = 7. **(B)** Colonic tissues isolated from littermate control mice or TAGAP-deficient mice were treated with HKLM for 3 or 6 h, followed by real-time PCR analysis of indicated genes. **(C)** Littermate control mice or TAGAP-deficient mice were oral gavaged with recombinant reg3g proteins daily for 1 week (5 μg/mouse/day), and feces were collected and analyzed by real time PCR for the indicated bacterial strains, *n* = 12, 8, 10 and 10. **(D,E)** Littermate control mice or TAGAP-deficient mice were oral gavaged recombinant reg3g proteins daily for 4 weeks (5 μg/mouse/day), followed by 2.5% DSS treatment for 5 days. Mice weight curve **(D)** and survival curve **(E)** were done in separate experiments, *n* = 7. **(F,G)** Littermate control mice or TAGAP-deficient mice were intraperitoneal injected anti-control MAb or anti-p40 MAb at day 1, 3, 5 and 7 after DSS treatment (100 μg/100 μl 1 × PBS/mouse). Mice weight curve **(F)** and survival curve **(G)** were done in separate experiments, *n* = 6. **(H)** Model of TAGAP regulating gut microbiota and Th17 cells abundance in the gut, and affecting IBD susceptibility. *p < 0.05, **p < 0.01, ***p < 0.001, ****p < 0.0001 based on 2-way ANOVA **(D,F)**, Log-rank (Mantel-Cox) Test for panels **(E,G)** and unpaired T test **(A–C)**. Data are representative of three independent experiments.

As antimicrobial peptide (AMP) plays an essential role in the gut homeostasis, next, we examined whether TAGAP deficiency led to decreased production of gut AMP. Indeed, we found that the expression of reg3g, an abundant and critical AMPs in the healthy gut, was significantly lower in colons from *Tagap^−/−^* mice compared to that of controls (Figure 6A). This reduced reg3g expression held true in resting conditions as well as following DSS-induced mucosal injury (Figure 6A). Moreover, we found that HKLM-induced induction of reg3g and reg3b was nearly abolished in colonic tissue from *Tagap^−/−^* mice (Figure 6B). Next, we examined whether recombinant reg3g has any bactericidal effect against *A. muciniphila* and *B. acidifaciens*. Under resting conditions, freshly-obtained fecal material from *Tagap^−/−^* mice contained significantly more *B. acidifaciens* than did fecal material from control mice (Figure 6C). Notably, oral administration of recombinant reg3g protein significantly decreased the abundance of *B. acidifaciens* in both control and *Tagap^−/−^* mice (Figure 6C). This result indicates that recombinant reg3g has a bactericidal effect against *B. acidifaciens*, and the dysregulation of gut microbiota and increased abundance of *B. acidifaciens* in TAGAP-deficient mice may be partially due to a defect in reg3g expression. We then tested whether the administration of recombinant reg3g protein has any therapeutic effect in the DSS-induced colitis model. Indeed, oral administration of recombinant reg3g rescued the DSS-induced systemic disease’s phenotype seen in both control and *Tagap^−/−^* mice (Figures 6D,E). This result indicates that recombinant reg3g have therapeutic potential for the treatment of IBD, as a medicine for the treatment of human IBD. Similarly, IL-12p40 neutralizing antibodies treatment have been reported to be effective in intestinal inflammation (Neurath et al., 1996; Becker et al., 2006; Fuss et al., 2006). Ustekinumab, an anti-p40 monoclonal antibody (MAb), has been shown in clinical trials to be a highly effective treatment for Crohn’s disease, and is FDA-approved for this indication (Niederreiter et al., 2013). However, despite this success ustekinumab fails to induce remission in approximately 30–40% of patients, and no validated biomarkers have been identified to predict clinical response. Given that *Tagap^−/−^* mice had significantly increased accumulation of colitogenic IL-17A-secreting CD4^+^ T cells in the colonic lamina propria, we tested whether the anti-p40 antibody has any therapeutic effect in TAGAP-deficient mice. Inhibition of p40 dramatically reduce DSS-induced systemic disease’s phenotype in *Tagap^−/−^* mice (Figures 6F,G). However, in *Tagap^+/−^* mice this therapeutic effect was markedly attenuated, and did not reach significance (Figures 6F,G). Together, this data suggests that p40 blockage might also work for colitis patients who carries TAGAP polymorphism rs212388^C^.

## Discussion

The TAGAP gene locus is associated with susceptibility to several autoimmune and inflammatory disorders, including MS and IBD. Our previous study finds that TAGAP plays a critical role in the antifungal innate immune response, and regulates peripheral T helper cells differentiation, which partially clarify the mechanism of TAGAP polymorphism to multiple sclerosis susceptibility (Chen et al., 2020). However, the mechanism of TAGAP polymorphism to IBD is still unknown. In the present study, we showed that TAGAP plays an important role in the production of AMPs in the gut, specifically reg3g. The absence of TAGAP-dependent reg3g production predisposed to gut dysbiosis, which was marked by loss of microbial diversity and new prominence of the commensals *A. muciniphila* and *B. acidifaciens*. Both *A. muciniphila* and *B. acidifaciens* rapidly induced the expression of pro-inflammatory cytokines in the lamina propria (particularly IL-1β, IL-6, and IL-23), which in turn promoted the local accumulation of colitogenic CD4^+^ T cells in the colon. This sequence of events resulted in colonic immune dysregulation that greatly exacerbated colitis disease activity following transient mucosal injury (Figure 6H). We also showed that this sequence can be disrupted - and colitis severity greatly attenuated - either by restoring gut microbiome homeostasis (*via* replacement of gut reg3g), or by directly attenuating the pathogenic function of the colitogenic CD4^+^ T cell population (*via* p40 inhibition). Consistent with this, reg3g complement effectively attenuated colitis severity in TAGAP-deficient mice. Moreover, we found that homozygosity for the common intronic variant rs212388^C^, which GWAS studies have identified as an IBD susceptibility locus, results in markedly reduced TAGAP gene expression in human cells. Therefore, the consequences of TAGAP deficiency, which we have now elucidated in the mouse, may also explain, at least in part, the mechanism by which rs212388^C/C^ confers susceptibility to IBD in humans. By extension, this also suggests that rs212388^C/C^ could hold promise as a biomarker for precision medicine-based approaches to IBD therapy selection and development. Here, we mainly explored the role of TAGAP in controlling gut bacterial communities, while we cannot exclude a possible role of TAGAP in regulating fungal communities in the gut, as we and others have found that TAGAP is involved in the regulation of anti-fungal immunity (Chen et al., 2020), and intestinal fungal communities also played a critical role in the pathogenesis of colitis.

Previous studies have described an important role for CD4^+^ T cells in the maintenance of gut homeostasis, and the dysregulation of gut CD4^+^ T cell responses have been shown to exacerbate colitis severity. In fact, one intriguing study recently demonstrated that that human-to-mouse fecal transplant using IBD patient donors promoted a dysbiotic pro-inflammatory colon environment, which was at least in part mediated by the activation and expansion of commensal antigen-specific Th17 cells (Britton et al., 2019). In the present study, we found that after DSS-induced mucosal injury in *Tagap^−/−^* mice, expression of the key pro-inflammatory cytokines IL-1β, IL-6, and IL-23 was highly induced in the gut. This resulted in markedly enhanced accumulation of IL-17-and IFN-γ-secreting CD4^+^ T cells in the colon of *Tagap^−/−^* mice compared to controls. We also demonstrated that inhibition of IL-12/IL-23-mediated signaling, using anti-p40 antibodies, was sufficient to attenuate colitis severity in TAGAP-deficient mice, suggesting that colitogenic CD4^+^ T cells critically mediate the severe colitis phenotype seen in TAGAP deficiency. Importantly, late phase clinical trials have already demonstrated the efficacy of T cell-directed therapies (including anti-p40, anti-a4b7, and JAK inhibitor) in Crohn’s disease, which further underscores the important role of T cells in IBD pathogenesis in humans.

*Akkermansia muciniphila* is an intestinal bacterium associated with wide-ranging host effects, including on host metabolism and the effectiveness PD-1 checkpoint immunotherapy. It has also been found to be associated with multiple disease states (Derrien et al., 2004; Cekanaviciute et al., 2017; Derrien et al., 2017). Recently, *A. muciniphila* was reported to induce immunoglobulin G1 (IgG1) antibody production and antigen-specific T cell responses in mice (Ansaldo et al., 2019). A small number of early-phase clinical trials have suggested that supplementation with *A. muciniphila* improves several metabolic parameters in overweight/obese insulin-resistant volunteers (Depommier et al., 2019). However, other recent reports have suggested an increase in *A. muciniphila* abundance in Parkinson’s disease (PD), multiple sclerosis (MS) and Alzheimer’s disease (AD) patients compared to controls (Cekanaviciute et al., 2017; Cirstea et al., 2018; Heintz-Buschart et al., 2018). These findings suggest that *A. muciniphila* may have unforeseen deleterious consequences for neurological health in certain individuals, which may be mediated by exacerbated neuroinflammatory responses. In the present study, we find that TAGAP-deficient mice have less abundance of *A. muciniphila* in the gut compared to control mice, which promotes us thinking that this strain may be a beneficial bacterial strain at beginning. While surprisingly, our later data indicates that *A. muciniphila* aggravate the severity of systemic disease associated with the DSS-induced model, primarily by promoting the activation and effector function of colitogenic CD4^+^ T cells. So, it seems that there is a contradiction between the less abundance of *A. muciniphila* in the TAGAP-deficient mice and more severe phenotype of TAGAP-deficient mice in colitis model. While, as we know, the severe phenotype and the hyper colitogenic CD4^+^ T cell in the TAGAP-deficient mice are come from the combined effect of multiple strains in the gut, and is impossible to only rely on one of the strains. Besides, *A. muciniphila* induces higher level of Th17 differentiation in TAGAP-deficient mice compared to that in the control mice, which could also compensate the colitogenic effect of the less abundance of *A. muciniphila* in TAGAP-deficient mice (Figure 5A).

Notably, *A. muciniphila* was able to induce colitogenic CD4^+^ T cell differentiation in TAGAP-deficient mice while Amuc_1100 did not, which indicates that the Amuc_1100-mediated impact on CD4^+^ T cells is TAGAP-dependent, while simultaneously suggesting that there are also other molecules in *A. muciniphila* capable of stimulating T cell response (Figures 5A–C). This is consistent with the finding that *A. muciniphila* aggravated the DSS-induced systemic disease’s phenotype, while Amuc_1100 alone did not (Figures 4E–H). One study by another group found that *A. muciniphila* can induce antigen-specific T cell responses, mainly through the outer membrane protein Amuc_RS03735. Our study indicates that Amuc_1100 may also be presented as an antigen and induce antigen-specific T cell responses under homeostatic conditions (Figure 5C), thus suggesting an important area for further investigation. A pathogenic role for *A. muciniphila* in colitis was previously reported in another study that employed the IL-10-deficient mouse model of colitis, while another study reported contradictory findings (Seregin et al., 2017; Ring et al., 2019). In the present study, we found that *A. muciniphila* can indeed aggravate DSS-induced systemic disease’s phenotype by promoting the differentiation and effector function of colitogenic CD4^+^ T cells in the gut, which highlights the need for additional investigation before further clinical trials utilizing this bacterium are undertaken.

## Materials and methods

### Human peripheral blood mononuclear cells study

Whole blood samples were drawn from each study participant. Genomic DNA was isolated using the Gentra Puregene blood (QIAGEN). All DNA samples were quantified using NanoDrop 2000 (Thermo Scientific, Wilmington, DE, United States) and inspected for quality by agarose gel electrophoresis. Human PBMCs were isolated by Ficoll-paque™ PREMIUM (17–5,442-02, GE Healthcare) from freshly drawn peripheral venous blood from healthy controls according to manufactory instruction. Briefly, add Ficoll-Paque media to the centrifuge tube, and layer the diluted blood sample onto the Ficoll-Paque media solution. Centrifuge at 400 g for 30 to 40 min at 18°C to 20°C with brake turned off. Draw off the upper layer containing plasma and platelets using a sterile pipette, leaving the mononuclear cell layer undisturbed at the interface. Wash the layer of mononuclear cells with 1 × PBS. This study followed the guidelines set forth by the Declaration of Helsinki and passed the review of the Ethics Committee of Sichuan Academy of Medical Sciences and Sichuan Provincial People’s Hospital. All study participants have signed a written informed consent form.

### Mice

The *Tagap* gene knockout mouse was a kind gift from Bernhard G Herrmann at Max Planck Institute for Molecular Genetics, Germany, and was reported in the original publication by Bauer et al. (Derrien et al., 2017). The accession number for the gene targeted in this knockout model is NM_145968, which corresponds to the *Tagap* gene. However, Bauer et al. refer to this gene as *Tagap1*. To clarify the gene targeted in these mice, we developed a quantitative reverse transcriptase-PCR method and showed that the targeted mice lacked *Tagap* mRNA, consistent with the accession number referenced in the original publication. See also MGI ID 3615484 for gene information and MGI ID 3603008 for mouse strain information. Experimental protocols were approved by the Institutional Animal Care and Use Committee of the Sichuan Provincial People’s Hospital.

### Reagents

Antibodies of anti-CD4 (GK1.5) and anti-IL-17A (TC11-18H10.1) were bought from Biolengend (cat no. 100406 and 506,908). Antibody of anti-IFN-γ (XMG1.2) was bought from eBioscience (cat no. 11–7,311-82). Control antibody (RG7/1.30) and Neutralizing anti-p40 (C17.8) antibody were bought from BioXcell (cat no. BE0251 and BE0051). HKLM was bought from Invivogen (cat no. tlrl-hklm). DSS (molecular weight, 40,000 kDa) was bought from MP Biomedicals. CD4^+^ T cell isolation kit was bought from Miltenyi Biotec (130–104-453). Bacterial strains of *A. muciniphila* and *B. acidifaciens* were bought from DSMZ (cat no. DSM 22959 and DSM 15896).

### Real-time PCR

Total RNA was extracted from cells or colonic samples with TRIzol (Invitrogen) according to the manufacturer’s instructions. 1 μg total RNA for each sample was reverse transcribed using the SuperScript® II Reverse Transcriptase from Thermo Fisher Scientific. The resulting complementary DNA was analyzed by real-time PCR using SYBR Green Real-Time PCR Master Mix. All gene expression results were expressed as arbitrary units relative to expression Actb or *GAPDH*. The Real-Time PCR primers sequence was shown in Table 1.

**Table 1:** Primer sequence.

Gene name	Forward primer	Reverse primer
*mIL-23*	5′-TCCTCCAGCCAGAGGATCACC-3′	5′-GCGCTGCCACTGCTGACTA-3′
*mIL-12*	5′-GCCAGTCCCGAAACCTGCTG-3′	5′-GCTGGTTTGGTCCCGTGTGA-3′
*mIL-2*	5′-CTGGAGCAGCTGTTGATGGA-3′	5′-TCAAATCCAGAACATGCCGC-3′
*mIL-6*	5′-GGACCAAGACCATCCAATTC-3′	5′-ACCACAGTGAGGAATGTCCA-3′
*mIL-1β*	5′-ATCTCGCAGCAGCACATCAA-3′	5′-ATGGGAACGTCACACACCAG-3′
*mCXCL1*	5′-TAGGGTGAGGACATGTGTGG-3′	5′-AAATGTCCAAGGGAAGCGT-3′
*mCXCL2*	5′-GTGAACTGCGCTGTCAATGC-3′	5′-GCTTCAGGGTCAAGGCAAAC-3′
*mTNFα*	5′-CAAAGGGAGAGTGGTCAGGT-3′	5′-ATTGCACCTCAGGGAAGAGT-3′
*mGM-CSF*	5′-CATCAAAGAAGCCCTGAACCTC-3′	5′-GTATGTCTGGTAGTAGCTGGCT-3′
*mReg3b*	5′-CTCTCCTGCCTGATGCTCTTAT-3′	5′-AGGCATAGCAGTAGGAGCCATA-3′
*mReg3g*	5′-GCCTATGGCTCCTATTGCTATG-3′	5′-CCACTGAGCACAGACACAAGAT-3′
*mPla2g2*	5′-CTCAATACAGGTCCAAGGGAAC-3′	5′-GTGGCATCCATAGAAGGCATAG-3′
*mLyz*	5′-GATGACATCACTGCAGCCATAC-3′	5′-GGGACAGATCTCGGTTTTGAC-3′
*mActin*	5′-GGTCATCACTATTGGCAACG-3′	5′-ACGGATGTCAACGTCACACT-3′

The primers for *A. muciniphila*, *B. acidifaciens* and general gut microbiota are listed below: 16S-A. muciniphila: 5′-cagcacgtgaaggtggggac-3′ and 5′-ccttgcggttggcttcagat-3′; 16S-*B. acidifaciens*: 5′-cacgtatccaacctgcctcat-3′ and 5′-tcatgcggtaggactatgacatc-3′; 16S: 5′-agagtttgatcctggctcag-3′ and 5′-ggttaccttgttacgactt-3′.

The specificity of primers was confirmed by using BLAST (NCBI).

### Dextran sodium sulfate-induced colitis model

Experimental colitis was induced by giving 2.5% or 3% (w/v) DSS (M.W. 40,000 kDa; MP Biomedicals Inc., Solon, OH) in drinking water. Mice (8 weeks) were treated for 5 days or 7 days, and then turned to normal water. For histological, gene expression, and cytokine production studies, mice were sacrificed after DSS treatment for indicated days. For evaluating the effect of *A. muciniphila*, *B. acidifaciens* and Amuc_1100 on DSS-induced colitis pathogenesis, mice were oral gavaged 1 × 10^8^ of each strain of bacterial or Amuc_1100 (10 μg/mouse) daily for 4 weeks, followed by the treatment of 2.5% of DSS for 5 days, and then changed to the normal water. The feces of mice were collected after antibiotics treatment, and real-time PCR was performed to verify the existence of the bacterial strains. Because of the accessibility reason, we did not use another microbiota bacterial as a negative control that did not modify the response. For anti-p40 MAb treatment experiment, 100 μg anti-control MAb or anti-p40 MAb were intraperitoneal (IP) injected into mice at day 2, 4, 6 and 8 after DSS treatment. For reg3g treatment experiment, mice were gavaged with 10 μg BSA or reg3g recombinant protein at day 2, day 4, day 6 and day 8 after the DSS treatment. For the clearance of gut microbiota, mice were pretreated for 2 weeks with antibiotics cocktail in the drinking water (ampicillin 1 mg/ml, neomycin 1 mg/ml, metronidazole 1 mg/ml, and vancomycin 0.5 mg/ml), and were changed to normal water for 2 days after antibiotics treatment, 3% of DSS water was given to the mice to set up colitis model. The feces of mice were collected after antibiotics treatment, and real-time PCR was performed to verify the clearance of gut microbiota. For observation of weight curve, euthanasia was performed for the mice when the condition of the mice was poor (curling up, trembling, do not eat, do not move, or weight below 70% of the start point).

### Fecal microbiota transplantation

Groups of 6–8 weeks old WT or TAGAP-deficient mice were administered a mixture of antibiotics in their drinking water for 2 weeks (ampicillin 1 mg/ml, neomycin 1 mg/ml, metronidazole 1 mg/ml, and vancomycin 0.5 mg/ml) to deplete the gut microbiota, and the feces were collected after antibiotics treatment, and real-time PCR was performed to verify the clearance of gut microbiota. Two days after stopping the antibiotic treatment, the mice were gavaged three times per week for 2 weeks with a fecal slurry made by homogenizing in PBS pooled cecal contents of donor mice. Two weeks after the fecal transplantation, the recipient mice were treated with 3% of DSS for 5 days as described above. Due to the breeding problem of the TAGAP-KO mice, we did not examine the composition of microbiota after fecal transplantation.

### Culture of *Akkermansia muciniphila* and *Bacteroides acidifaciens*

A basal medium (Brain-heart Infusion Extract, 37 g, Yeast Extract, 5 g, 0.1% Resazurin, 1 ml add water to 1 l). After autoclave, add 10 ml of filter-sterilized 10% (W/V) L-cysteine hydrochloride. *A. muciniphila* (ATCC BAA-835) was cultured anaerobically in a basal medium, supplemented with mucin (0.25%). *B. acidifaciens* (DSM 15896) was cultured anaerobically in a basal medium.

### 16S rDNA sequencing and microbial analysis

The 16S rDNA sequencing was performed by BGI Co., Ltd. Briefly, Microbial DNA from mice fecal samples was extracted using the TIANamp Stool DNA Kit (cat no. DP328, TIANGEN, Beijing, China) according to the manufacturer’s instructions. The 16S rDNA sequencing library was constructed following the 16S rDNA gene Metagenomic Sequencing Library Preparation Illumina protocol, targeting the variable regions 3 and 4 (V3–V4) which were amplified using the 341F and 806R primers (341F: 5′- ACTCCTACGGGAGGCAGCAG-3′ and 805R: 5’-GGACTACHVGGGTWTCTAAT-3′). The quality control was performed with bioanalyzer Agilent 2,100 (Agilent Technologies), then the qualified library was sequenced with Illumina HiSeq2500 platform. The sequences were filtered for quality and a mean of 32,215 reads per sample were retained with FLASH (Fast Length Adjustment of Short reads, v1.2.11). Reads were clustered into operational taxonomic units (OTUs; 97% identity threshold) with USEARCH (v7.0.1090), and representative OTU sequences were assigned using the RDP Classifier software (v.2.2), with a minimal confidence of 60%. Finally, a phylogenetic tree was constructed within the QIIME (v1.80) package using FastTree and filtered PyNAST alignments of the OTU representative sequences. To explore microbial diversity, alpha diversity (Shannon’s diversity index) and beta diversity (weighted and unweighted UniFrac) were calculated using MOTHUR software (v1.31.2) and QIIME (v1.80), respectively. The raw reads of 16S sequencing of gut microbiota DNA were submitted to the NCBI Sequence Read Archive (SRA) database (accession number: PRJNA874477).

### T cells polarization by *Akkermansia muciniphila*, *Bacteroides acidifaciens* and Amuc_1100

To examine T cell polarization by *A. muciniphila*, *B. acidifaciens* and Amuc_1100, littermate control mice or TAGAP-deficient mice were gavaged with live *A. muciniphila* (1 × 10^9^), *B. acidifaciens* (1 × 10^9^) or Amuc_1100 (10 μg) twice a week for 2 weeks, and colonic lamina propria cells were isolated and incubated with *A. muciniphila* (MOI = 50 or 100), *B. acidifaciens* (MOI = 50 or 100) or Amuc_1100 (2 μg or 10 μg) for another 3 days. Flow cytometry was performed by analyzing CD4^+^IL-17A or CD4^+^IFN-γ-producing cells.

### Purification of recombinant a.muc_1100 protein

The coding sequence of A.muc_1100 protein was constructed into pET28a plasmid which express a C-terminal His-tag. Recombinant A.muc_1100 was expressed and purified from *E. coli*. The BL21 (DE3) strain harboring a plasmid encoding A.muc_1100-His recombinant protein was induced with 0.5 mM isopropyl-β-D-thiogalactopyranoside (IPTG) at 22°C for 20 h. His-tagged A.muc_1100 was purified with His-tag Protein Purification Kit (Beyotime) according to the manufacturer’s instructions. Eluted A.muc_1100 protein was concentrated and buffer-exchanged to 1 × PBS buffer.

### Statistics

Statistical significance between two groups was determined by unpaired two-tailed T test; Multiple-group comparisons were performed using One-Way ANOVA; the weight change curve was analyzed by two-way ANOVA test for multiple comparisons. *p* < 0.05 was considered to be significant. Results are shown as mean and the error bar represents standard error of mean (S.E.M) technical as indicated in the figure legend. All the statistical analysis was done by using GraphPad Prism 8.02 software.

## Data availability statement

The datasets presented in this study can be found in online repositories. The names of the repository/repositories and accession number (s) can be found at: https://www.ncbi.nlm.nih.gov/, PRJNA874477.

## Ethics statement

The studies involving human participants were reviewed and approved by Sichuan Academy of Medical Sciences and Sichuan Provincial People’s Hospital. The patients/participants provided their written informed consent to participate in this study. The animal study was reviewed and approved by Sichuan Academy of Medical Sciences and Sichuan Provincial People’s Hospital.

## Author contributions

RH, JC, and ZZ did the experiments. CS, YD, MY, LF, QP, ZC, RG, HW, and CW contributed to the experiments. YH helped to get human PBMC samples. CS and ZL helped to culture the bacterial strains of *Akkermansia muciniphila* and *Bacteroides acidifaciens*. CW wrote the manuscript. CW and ZL oversaw the experiments. All authors contributed to the article and approved the submitted version.

## Funding

This investigation was supported by the grant from the Key Research and Development Program of Sichuan province (22ZDYF3738 to CW); the Postdoctoral Foundation of Sichuan Provincial People’s Hospital (2022BH01 to RH; 2022BH07 to MY); the Postdoctoral Grant from Chinese Postdoctoral Science Foundation (grant no. 2022M720659 to RH); the Postdoctoral Research Project of Sichuan Provincial Department of Human Resources and Social Security (grant no. TB2022086 to RH).

## Conflict of interest

The authors declare that the research was conducted in the absence of any commercial or financial relationships that could be construed as a potential conflict of interest.

## Publisher’s note

All claims expressed in this article are solely those of the authors and do not necessarily represent those of their affiliated organizations, or those of the publisher, the editors and the reviewers. Any product that may be evaluated in this article, or claim that may be made by its manufacturer, is not guaranteed or endorsed by the publisher.

## Footnotes

^1^ https://www.ncbi.nlm.nih.gov/snp/rs212388#frequency_tab
