# Supplementary material for: T-cell activation Rho GTPase-activating protein maintains intestinal homeostasis by regulating intestinal T helper cells differentiation through the gut microbiota
Source: Front Microbiol. 2023 Jan 10;13:1030947. doi: 10.3389/fmicb.2022.1030947 (PMC9873376; doi:10.3389/fmicb.2022.1030947)
Supplement: Supplementary file 1 [file Data_Sheet_1.docx]

Supplementary Material


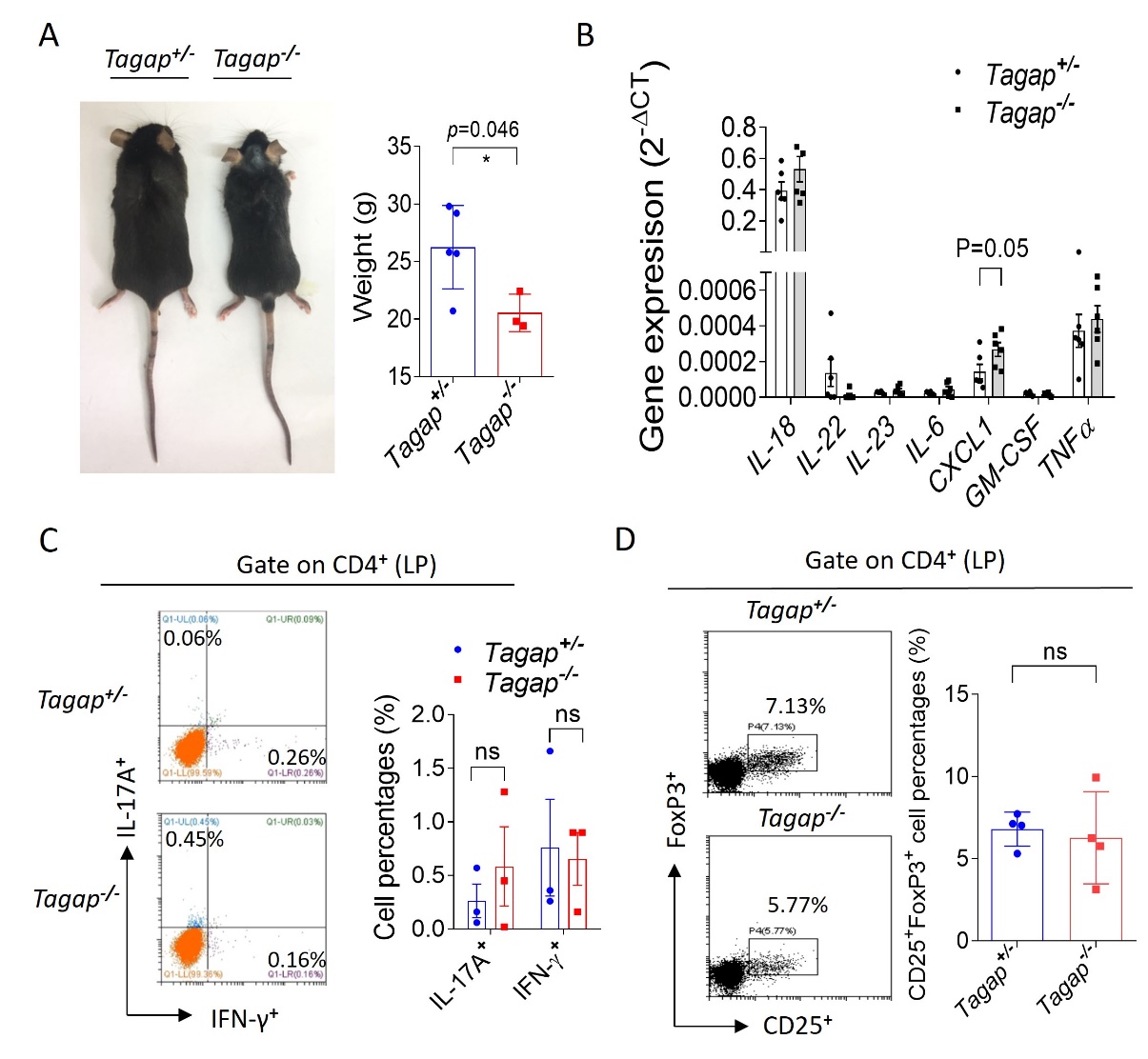


**Supplementary Figure 1. Phenotype of TAGAP-deficient mice. (A)** Representative image of 10 weeks old littermate control mice and TAGAP-deficient mouse were shown. Weight of littermate control mice and TAGAP-deficient mouse were shown on the right panel. **(B)** Colonic tissue from littermate control mice and TAGAP-deficient mouse were isolated, followed by real-time PCR analysis of indicated gene expression. n=6. **(C)** Cells were isolated from lamina propria of littermate control or TAGAP-deficient mice, followed by flow cytometry analysis of indicated cells. **(D)** Cells were isolated from colonic lamina propria from littermate control mice or TAGAP-deficient mice after DSS treatment for 5 days, followed by flow cytometry analysis of CD4**^+^**CD25**^+^**Foxp3**^+^** Treg cells. n=4. *: *P*<0.05, **: *P*<0.01, ***: *P*<0.001, ****: *P*<0.0001 based on two-sided unpaired T test (**A-D**). Data are representative of three independent experiments.


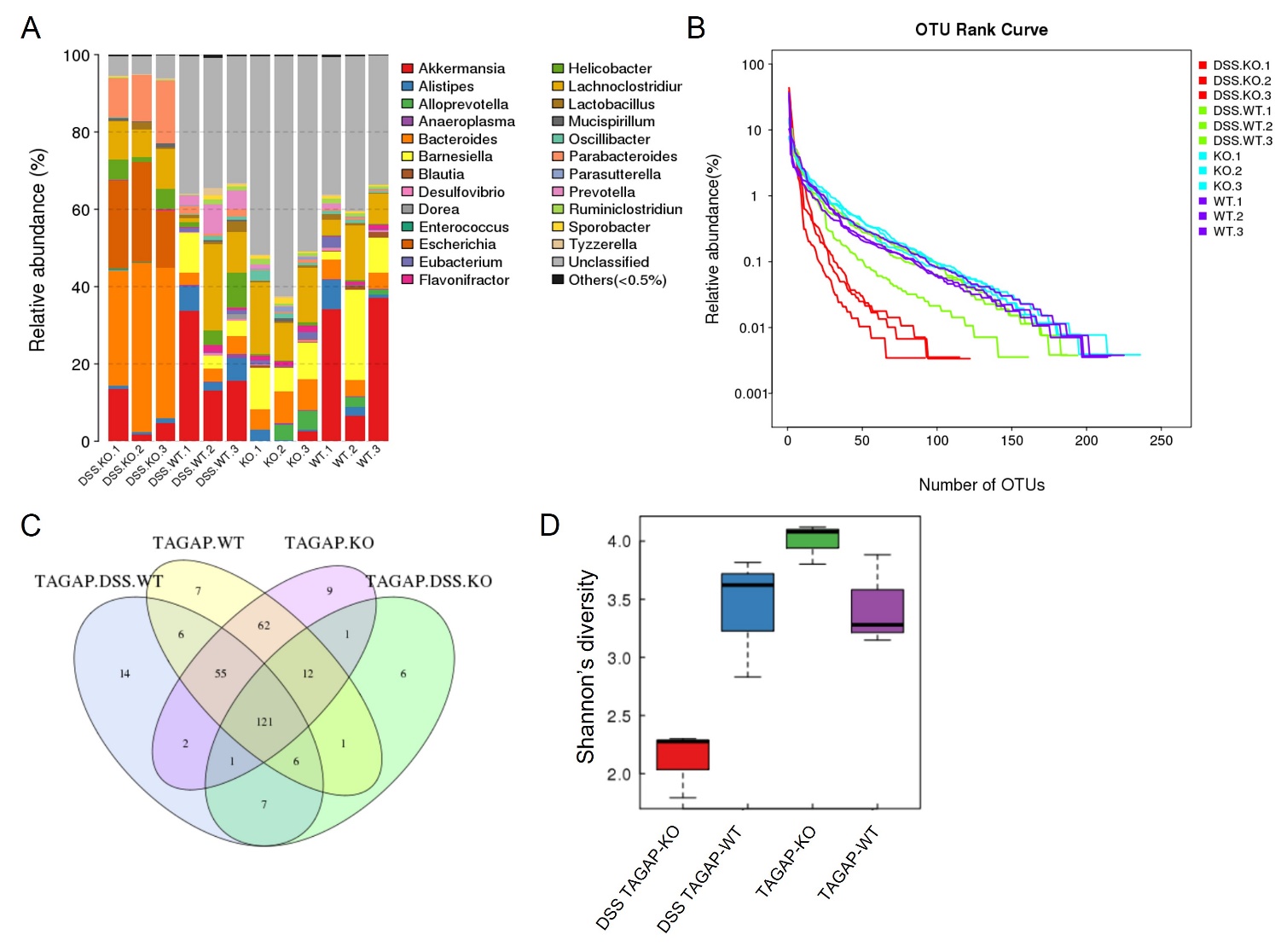


**Supplementary Figure 2. TAGAP-deficient mice had altered microbiota compared to control mice. (A)** Relative abundance of different bacterial genus in the feces of littermate control mice or TAGAP-deficient mice identified by 16S sequencing was shown. **(B)** Quantitative analysis of different bacterial strain in the feces of littermate control mice or TAGAP-deficient mice. n=3. **(C)** Venn diagram at the species level based on OTU numbers. Different colors represent different groups. The overlaps represent the common taxa between groups, and the non-overlapping portions represent unique taxa in each group. **(D)** Box plot based on alpha diversity (Shannon index) of the gut microbial communities of littermate control mice or TAGAP-deficient mice.


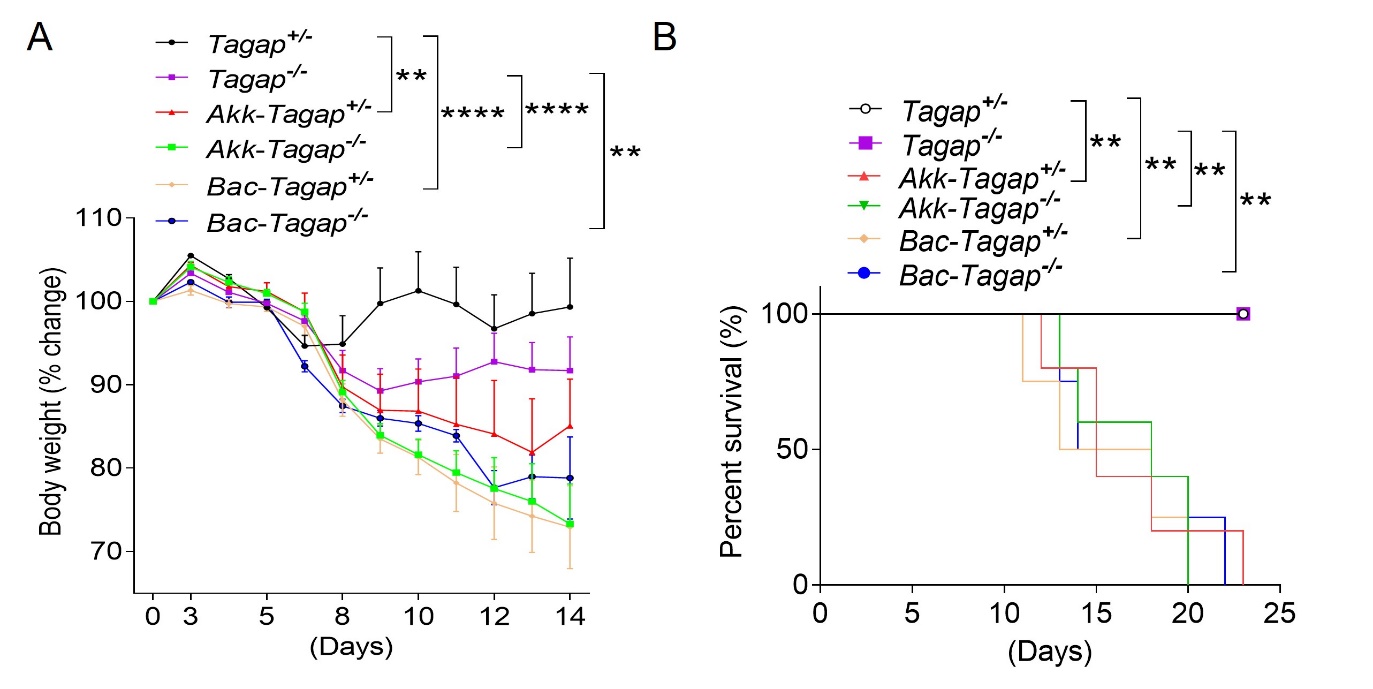


**Supplementary Figure 3. *A.* *muciniphila* or *B. acidifaciens* increased DSS-induced systemic disease’s severity in control mice and TAGAP-deficient mice. (A-B)** Littermate control mice or TAGAP-deficient mice were pretreated with antibiotics for two weeks as described in the Methods, and were changed to normal water for 2 days. Mice were then oral gavaged *A.* *muciniphila* and *B. acidifaciens* daily for 4 weeks (1×10^8^/mouse/day), followed by 2.5% DSS treatment for 5 days. The weight loss (**A**) and survival data (**B**) were from separate experiments. n=5. *: *P*<0.05, **: *P*<0.01, ***: *P*<0.001, ****: *P*<0.0001 based on Two-way ANOVA **(A)**, Log-rank (Mantel-Cox) Test for **(B)**. Data are representative of three independent experiments.
